# Utilization Trends and Predictors of Non-invasive and Invasive Ventilation During Hospitalization Due to Community-Acquired Pneumonia

**DOI:** 10.7759/cureus.17954

**Published:** 2021-09-14

**Authors:** Harshil Shah, Jude ElSaygh, Abdur Raheem, Mohammed A Yousuf, Lac Han Nguyen, Pratiksha S Nathani, Venus Sharma, Abhinay Theli, Maheshkumar K Desai, Dharmeshkumar V Moradiya, Hiteshkumar Devani, Apurwa Karki

**Affiliations:** 1 Internal Medicine, Guthrie Robert Packer Hospital, Sayre, USA; 2 Internal Medicine, University of Debrecen, Debrecen, HUN; 3 Internal Medicine, Texas Tech University Health Sciences Center at Permian Basin, Odessa, USA; 4 General Medicine, Gleneagles Global Hospitals, Hyderabad, IND; 5 Internal Medicine, University of Medicine and Pharmacy of Ho Chi Minh City, Ho Chi Minh City, VNM; 6 Internal Medicine, Maharashtra University of Health Sciences, Nashik, IND; 7 Internal Medicine, Punjab Institute of Medical Sciences, Jalandhar, IND; 8 Internal Medicine, Guthrie Cortland Medical Center, Cortland, USA; 9 Internal Medicine, Hamilton Medical Center, Medical College of Georgia/Augusta University, Augusta, USA; 10 Internal Medicine, St John of God Murdoch Hospital, Murdoch, AUS; 11 Dental Medicine, University of Pittsburgh School of Dental Medicine, Pittsburgh, USA; 12 Critical Care, Guthrie Cortland Medical Center, Cortland, USA

**Keywords:** outcomes, trends, invasive mechanical ventilation, non-invasive ventilation, community acquired pneumonia

## Abstract

Background: Community-acquired pneumonia (CAP) is associated with significant morbidity and mortality. Non-invasive ventilation (NIV) and invasive mechanical ventilation (IMV) are most important interventions for patients with severe CAP associated with respiratory failure. We analysed utilization trends and predictors of non-invasive and invasive ventilation in patients hospitalized with CAP.

Methods: Nationwide Inpatient Sample and Healthcare Cost and Utilization Project data for years 2008-2017 were analysed. Adult hospitalizations due to CAP were identified by previously validated International Classification of Diseases, 9th Revision, Clinical Modification (ICD-9-CM) and International Classification of Diseases, 10th Revision, Clinical Modification (ICD-10-CM) codes. We then utilized the Cochran-Armitage trend test and multivariate survey logistic regression models to analyse temporal incidence trends, predictors, and outcomes. We used SAS 9.4 software (SAS Institute Inc., Cary, NC, USA) for analysing data.

Results: Out of a total of 8,385,861 hospitalizations due to CAP, ventilation assistance was required in 552,395 (6.6%). The overall ventilation use increased slightly; however, IMV utilization decreased, while NIV utilization increased. In multivariable regression analysis, males, Asian/others and weekend admissions were associated with higher odds of any ventilation utilization. Concurrent diagnoses of septicemia, congestive heart failure, alcoholism, chronic lung diseases, pulmonary circulatory diseases, diabetes mellitus, obesity and cancer were associated with increased odds of requiring ventilation assistance. Ventilation requirement was associated with high odds of in-hospital mortality and discharge to facility.

Conclusion: The use of NIV among CAP patients has increased while IMV use has decreased over the years. We observed numerous factors linked with a higher use of ventilation support. The requirement of ventilation support is also associated with very high chances of mortality and morbidity.

## Introduction

Community-acquired pneumonia (CAP) refers to acute pulmonary parenchymal inflammation in a person who has not been hospitalized in the previous 14 days or is not living in a nursing home or long-term care facility [1]. CAP is estimated to occur in five million cases in the USA, leading to over one million hospitalizations and 60,000 deaths each year [2].

The indications of ventilation in CAP remain controversial. The American Thoracic Society and the Infectious Disease Society of America suggested cautious trials of non-invasive ventilation (NIV) while its application is still not a recommendation provided by the evidence-based clinical practice guidelines of CAP [3]. A study conducted in 2018 showed that the 30-day mortality was 33%, 16% and 6% in invasive mechanical ventilation (IMV), NIV and non-ventilation groups, respectively [4]. Based on this study, the use of IMV can be considered as an independent predictor of mortality in severe CAP patients.

Most of previous research studies on ventilation use among CAP patients were conducted on relatively small sample sizes or in single centers. Therefore, lack of generalized statistics from large sample size impedes the accurate acknowledgement of utilization trends, predictors and outcomes. We used a large, nationally representative database to describe temporal trends, predictors and outcomes of invasive and non-invasive ventilation use in patients with CAP.

## Materials and methods

Data source

We extracted our study cohort from the National (Nationwide) Inpatient Sample (NIS) of the Healthcare Cost and Utilization Project (HCUP), Agency for Healthcare Research and Quality (AHRQ) [5]. NIS is one of the largest all-payer publicly available databases on inpatient discharges from U.S. hospitals maintained by the AHRQ [5]. The NIS approximates a 20% stratified sample of discharges from U.S. community hospitals, excluding rehabilitation and long-term acute care hospitals and contains more than seven million hospitalizations annually. With the established weights in NIS, this data could be weighted to represent the standardized U.S. population and obtain national estimates with high accuracy [6].

Study population and design

We queried the 2008-2017 NIS database using International Classification of Diseases, 9th Revision, Clinical Modification, and International Classification of Diseases, 10th Revision, Clinical Modification (ICD-9/10-CM) diagnose codes for CAP. These codes have been used by previously published articles from administrative databases such as NIS. We also identified IMV and NIV by ICD-9/10-CM procedural codes [7-11]. We extracted demographics, hospital-level characteristics (geographical region, size, and teaching status) and patient-level characteristics from the NIS [12]. We estimated comorbidities using Elixhauser Comorbidity Software and mortality risk using the validated All Patient Refined-Diagnosis Related Group (APR-DRG) Risk of Mortality score, which are also supplied by HCUP tools and software [13,14]. Specific concurrent medical conditions and procedures of interest were identified by ICD-9/10-CM diagnosis and procedure codes.

Statistical analysis

To establish the trend, we calculated the proportion of CAP hospitalizations that required IMV or NIV for each year and used the Cochran-Armitage trend test for analysis purpose. Descriptive statistics were performed to present the baseline difference in socio-demographics, comorbidities and hospital-level characteristics among those who did not require any ventilation, those who required IMV and those who required NIV. Categorical variables were compared with the chi-square test, and continuous variables were compared with Student's t-test or Wilcoxon rank-sum test. To estimate the impact of IMV or NIV on CAP outcomes, we used logistic outcomes/variables (in-hospital mortality and discharge to a long-term facility) and adjusted them for potential confounders. We utilized SAS 9.4 (SAS Institute Inc., Cary, NC, USA) for all analyses and included designated weight values to produce nationally representative estimates [6]. For regression models, we used survey procedures to account for the inherent survey design of NIS to produce more robust estimates [15]. We considered a two-tailed p-value <0.05 as statistically significant.

## Results

We analysed a total of 8,385,861 hospitalizations due to CAP from 2008 to 2017. Out of the total, 267,774 (3.2%) required NIV and 284,621 (3.4%) required IMV during the hospitalization.

Temporal trends of NIV and IMV utilization during CAP hospitalizations

**Figure 1:**
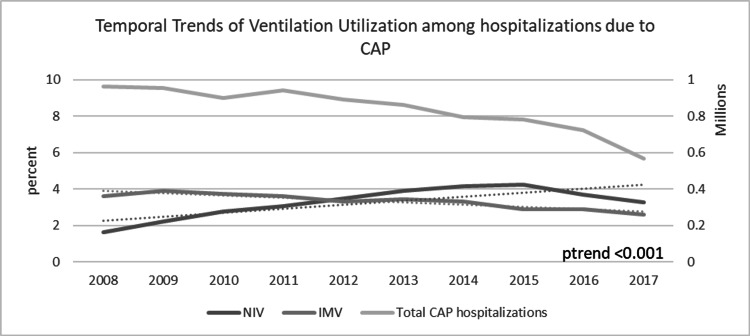
Temporal trends of NIV and IMV utilization during CAP hospitalizations CAP, community-acquired pneumonia; NIV, non-invasive ventilation; IMV, invasive mechanical ventilation

In trend analysis, we observed steady decline in yearly CAP hospitalizations from 963,170 in 2008 to 568,210 in 2017. The proportion of patients receiving NIV was increased from 1.7% in 2008 to 3.3% in 2017, while the trend of IMV declined from 3.6% in 2008 to 2.6% in 2017 (Figure 1).

Baseline characteristics of the study cohort

The median age (IQR) was 71 (56-81), 71 (60-80) and 66 (55-77) years in patients who received no ventilation, NIV and IMV, respectively. The proportion of females was higher among those who received NIV (52.5% vs 47.5%; p<0.001); however, the proportion of males was higher among the those who received IMV (52.0% vs 47.9%; p<0.001). White patients comprised 69.1% of total CAP hospitalizations followed by African Americans (10.5%) but the proportion of African Americans was 13.4% among those who required IMV. In patients who received NIV, the most common comorbidities were chronic pulmonary disease (68.5%), hypertension (68.4%), fluid and electrolyte abnormalities (45.4%) and congestive heart failure (42.8%). In patients who received IMV, the most common comorbidities were fluid and electrolyte disorders (59.9%), hypertension (56.4%), and chronic pulmonary disease (46.8%). The northeast region had a higher proportion of NIV (22.4%) and IMV (19.8%) as compared to no ventilation (17.5%) (p<0.001). A similar trend was observed in large-bed-size hospitals and urban teaching hospitals. A detailed description of baseline characteristics of the study cohort has been depicted in Table 1.

**Table 1:** Baseline characteristics of the study cohort HMO, Health Maintenance Organization ^†^Based on the^ ^quartile classification of the estimated median household income of residents in patients' zip code

Patient and hospital characteristics	No ventilation	Non-invasive ventilation	Invasive mechanical ventilation	Total	p value
Overall	7,833,466	267,774	284,621	8,385,861	
Age in years (mean±SE)	68.4 (0.5)	69.6 (0.8)	65.2 (0.1)		<0.001
Age in years (median [q1-q3])	71 (56-81)	71 (60-80	66 (55-77)		<0.001
Age in years (%)					<0.001
18-34	5.0	2.6	5.6	4.98	
35-49	9.9	7.1	10.3	9.77	
50-64	21.7	23.3	28.0	21.96	
65-79	31.2	37.8	35.2	31.52	
≥80	32.2	29.2	21.0	31.76	
Gender (%)					<0.001
Male	46.8	47.5	52.0	47.03	
Female	53.1	52.5	47.9	52.94	
Race (%)					<0.001
White	69.2	72.0	64.3	69.09	
Black	10.4	10.4	13.4	10.51	
Hispanic	6.9	6.8	8.2	6.89	
Others	4.6	4.6	5.4	4.58	
Missing	9.0	6.2	8.8	8.93	
Comorbidities (%)					
Obesity	10.2	23.1	14.2	10.72	<0.001
Hypertension	61.2	68.4	56.4	61.22	<0.001
Diabetes mellitus with chronic complications	6.6	10.1	8.4	6.8	<0.001
Diabetes mellitus without chronic complications	22.7	27.8	24.4	22.88	<0.001
Congestive heart failure	22.9	42.8	37.2	24.01	<0.001
Valvular heart disease	7.0	9.9	8.6	7.11	<0.001
History of chronic pulmonary disease	45.9	68.5	46.8	46.66	<0.001
Pulmonary circulatory disease	4.6	11.9	9.8	4.99	<0.001
Peripheral vascular disease	6.5	8.7	7.0	6.56	<0.001
Paralysis	2.8	3.6	7.4	2.94	<0.001
Coagulopathy	4.8	6.7	12.9	5.15	<0.001
Solid tumor without metastasis	3.8	4.1	4.6	3.88	<0.001
Lymphoma	1.8	1.6	2.4	1.84	<0.001
Metastatic cancer	3.5	4.0	4.9	3.58	<0.001
Weight loss	6.9	9.3	19.7	7.39	<0.001
Liver disease	2.8	2.8	4.7	2.87	<0.001
Alcoholism	2.8	2.7	5.4	2.86	<0.001
Neurology disorders	11.9	12.1	15.4	11.99	<0.001
Renal failure	17.6	23.4	22.4	17.91	<0.001
Hypothyroidism	15.2	16.1	12.8	15.11	<0.001
Arthritis	4.3	4.1	4.1	4.28	<0.001
Anemia deficiency	25.2	28.8	34.4	25.59	<0.001
Blood loss	0.6	0.8	1.2	0.64	<0.001
Fluid and electrolyte disorders	34.7	45.4	59.9	35.88	<0.001
Depression	13.1	15.7	11.3	13.15	<0.001
Psychoses	5.0	6.4	6.1	5.07	<0.001
Drug abuse	2.7	2.7	3.7	2.76	<0.001
Peptic ulcer disease	0.1	0.2	0.2	0.14	<0.001
AIDS	0.0	0.0	0.1	0.02	<0.001
Median household income (%)^†^					<0.001
1st quartile	32.0	29.6	32.2	31.91	
2nd quartile	27.2	26.8	25.7	27.13	
3rd quartile	21.8	22.7	22.1	21.81	
4th quartile	16.8	18.8	17.5	16.91	
Primary insurance (%)					<0.001
Medicare/Medicaid	76.1	82.3	77.3	76.33	
Private including HMO	17.5	13.5	17.0	17.35	
Uninsured/self-pay	6.2	4.1	5.5	6.13	
Hospital bed size (%)					<0.001
Small	21.6	16.1	13.2	21.16	
Medium	27.3	29.2	27.1	27.31	
Large	50.7	54.5	59.0	51.12	
Hospital type (%)					<0.001
Rural	22.0	15.2	12.1	21.4	
Urban non-teaching	38.6	39.0	38.5	38.59	
Teaching	39.1	45.6	48.8	39.6	
Hospital region (%)					<0.001
Northeast	17.5	22.4	19.8	17.72	
Midwest	25.1	20.2	21.9	24.86	
South	41.0	40.8	40.0	40.96	
West	16.4	16.7	18.3	16.46	
Day of admission					<0.001
Weekday	75.0	73.7	74.2	74.96	
Weekend	25.0	26.3	25.8	25.04	
Source of admission (%)					<0.001
Transfer from other hospital or other health facility	20.6	12.7	19.0	20.26	
Emergency department	79.4	87.3	81.0	79.74	
Type of admission (%)					<0.001
Emergent or urgent	92.8	96.3	95.0	92.96	
Elective	7.2	3.7	5.0	7.04	

Predictors of ventilation utilization during CAP hospitalizations

From our analysis, several predictors associated with increased ventilation utilization (a composite of either NIV or IMV) were the age group 50-64 (OR 1.1; 95% CI 1.06-1.15; p<0.0001), obesity (OR 1.86; 95% CI 1.82-1.90; p<0.0001), septicemia (OR 6.14; 95% CI 5.98-6.30; p<0.0001), congestive heart failure (OR 2.15; 95% CI 2.12-2.19; p<0.0001), history of chronic pulmonary disease (OR 1.61; 95% CI 1.58-1.64; p<0.0001), pulmonary circulatory disease (OR 1.83; 95% CI 1.79-1.88; p<0.0001), paralysis (OR 1.94; 95% CI 1.86-2.01; p<0.0001), and electrolyte and fluid disorders (OR 1.88; 95% CI 1.85-1.92; p<0.0001).

Meanwhile, we also found several predictors associated with reduced ventilation utilization: age >80 (OR 0.66; 95% CI 0.63-0.69; p<0.0001), female sex (OR 0.89; 95% CI 0.88-0.90; p<0.0001), uninsured/self-pay (OR 0.86; 95% CI 0.83-0.89; p<0.0001), small hospital bed size (OR 0.66; 95% CI 0.64-0.69; p<0.0001), and rural hospital (OR 0.60; 95% CI 0.57-0.63; p<0.0001). Detailed predictors of ventilation utilization are shown in Table 2.

**Table 2:** Predictors of ventilation utilization during CAP hospitalizations CAP, community-acquired pneumonia; HMO, Health Maintenance Organization

Independent variable/characteristic	Odd ratio	95% CI (LL)	95% CI (UL)	p value
Year	1.02	1.01	1.02	0.0001
Age in years				
18-34	ref	ref	ref	
35-49	0.97	0.93	1.01	0.1085
50-64	1.10	1.06	1.15	0.0001
65-79	0.98	0.94	1.02	0.2907
≥80	0.661	0.632	0.691	0.0001
Gender				
Male	ref	ref	ref	
Female	0.89	0.88	0.90	0.0001
Race				
White	ref	ref	ref	0.0001
Black	0.956	0.928	0.985	0.0028
Hispanic	1.025	0.986	1.065	0.2107
Others	1.082	1.041	1.125	0.0001
Comorbidities/concurrent diagnosis				
Obesity	1.86	1.82	1.90	0.0001
Hypertension	0.93	0.91	0.95	0.0001
Septicemia	6.14	5.98	6.30	0.0001
Diabetes mellitus	1.03	1.01	1.06	0.0181
Congestive heart failure	2.15	2.12	2.19	0.0001
Valvular heart disease	1.02	1.00	1.05	0.0967
History of chronic pulmonary disease	1.61	1.58	1.64	0.0001
Pulmonary circulatory disease	1.83	1.79	1.88	0.0001
Peripheral vascular disease	1.02	0.99	1.04	0.2353
Paralysis	1.94	1.87	2.01	0.0001
Metastatic cancer	1.19	1.15	1.24	0.0001
Weight loss	1.82	1.77	1.87	0.0001
Liver disease	0.99	0.96	1.03	0.7755
Alcoholism	1.23	1.18	1.28	0.0001
Anemia deficiency	1.12	1.10	1.14	0.0001
Drug abuse	1.01	0.97	1.05	0.6015
Neurological disorders	1.24	1.22	1.27	0.0001
Renal failure	1.03	1.01	1.05	0.0053
Arthritis	0.95	0.91	0.98	0.0011
Electrolyte and fluid disorders	1.88	1.85	1.92	0.0001
Lymphoma	1.03	0.97	1.08	0.3853
Median household income				
1st quartile	0.96	0.93	1.00	0.0352
2nd quartile	0.98	0.95	1.02	0.3741
3rd quartile	0.98	0.95	1.01	0.1998
4th quartile	ref	ref	ref	
Primary Insurance				
Medicare/Medicaid	ref	ref	ref	
Private including HMO	0.60	0.57	0.63	0.0001
Uninsured/self-pay	0.86	0.83	0.89	0.0001
Hospital bed size				
Small	0.66	0.64	0.69	0.0001
Medium	0.90	0.86	0.93	0.0001
Large	ref	ref	ref	
Hospital type				
Rural	0.60	0.57	0.63	0.0001
Urban non-teaching	0.86	0.83	0.89	0.0001
Teaching	ref	ref	ref	
Hospital region				
Northeast	1.24	1.18	1.30	0.0001
Midwest	0.82	0.77	0.87	0.0001
South	0.98	0.94	1.02	0.3569
West	ref	ref	ref	
Day of admission				
Weekday	ref			
Weekend	1.05	1.03	1.06	0.0001
Source of admission				
Transfer from other hospital or other health facility	ref	ref	ref	
Emergency department	0.92	0.89	0.95	0.0001
Type of admission				
Emergent or urgent	ref	ref	ref	
Elective	0.84	0.80	0.90	0.0001

Outcomes of ventilation utilization during CAP hospitalizations

We divided all the CAP hospitalizations into those who received no ventilation, those who received NIV and those who received IMV. There was a comparable difference between the patterns of discharge disposition in patients who received no ventilation, NIV or IMV. Among those patients who did not receive any ventilation, we found that 74.6% were discharged home, 23.2% discharged to a facility and 2.2% died during the hospital stay. This mortality was significantly different from those patients who received either NIV or IMV, with 10.7% mortality for the NIV group and 29.8% for the IMV group. In the NIV group, 56.7% went home and 32.5% were discharged to a facility. In the IMV group, 28.6% were discharged home, while 41.6% were discharged to a facility. Even after adjusting with confounders, we found that any ventilation utilization was associated with higher adjusted in-hospital mortality (OR 10.4; 95% CI 10.09-10.67, p<0.001) and discharge to facility (OR 2.05; 95% CI 2.0-2.09, p<0.001). Length of stay (LOS) was the lowest among the 'no ventilation' group with 5 days. The mean LOS for those who required NIV was 8 days, while that for the IMV group was the highest, at 15 days (p<0.001).

**Figure 2:**
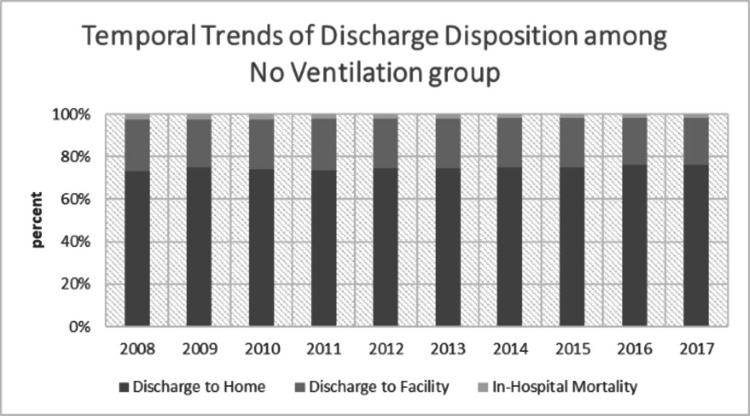
Temporal trends of discharge disposition among the no ventilation group

**Figure 3:**
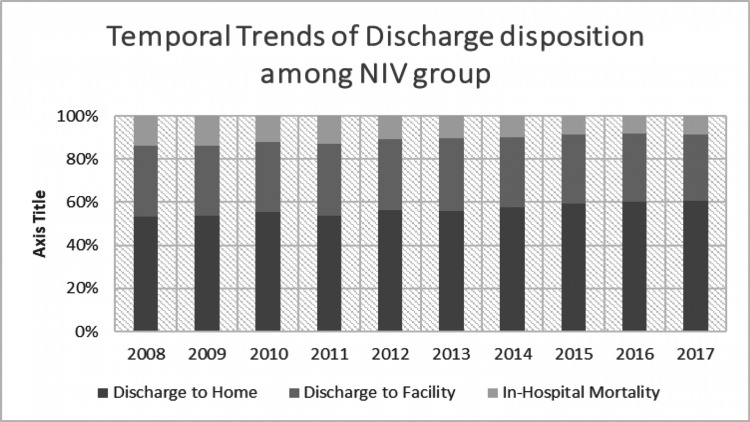
Temporal trends of discharge disposition among the NIV group NIV, non-invasive ventilation

**Figure 4:**
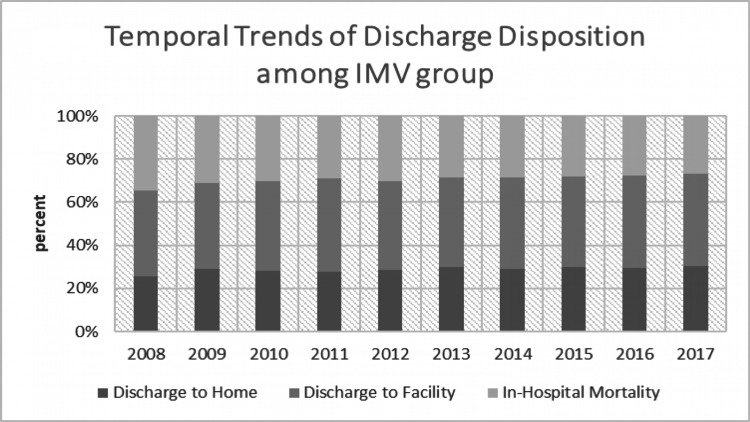
Temporal trends of discharge disposition among the IMV group IMV, invasive mechanical ventilation

In trend analysis, in-hospital mortality declined from 2.8% in 2008 to 1.7% in 2017 (p<0.001) among those who did not require any sort of ventilation. A similar trend was observed in NIV (14% in 2008 to 8.5% in 2017; p<0.001) and IMV (34.8% in 2008 to 26.7% in 2017; p<0.001) groups. Detailed visualization of discharge disposition trends from 2008 to 2017 of CAP hospitalization among all three groups has been presented in Figures 2-4.

## Discussion

In our study, the total hospitalizations due to CAP are declining from 2008 to 2017. However, a study done on CAP in Spain reported a significant increase in hospitalizations due to CAP between 2003 and 2014 [16]. Another study done in Denmark stated that total pneumonia hospitalizations increased by 63%, between 1997 and 2011. In our study period of 2008 to 2018, an increase of NIV use in CAP was seen while the trend of IMV use in CAP declined. A study analysing CAP hospitalizations in Spain between 2001 and 2015 also showed that the use of NIV and NIV+IMV increased significantly over the time period specified while the IMV utilization decreased [17,18].

The results of our study suggest that the age range 50-64 is associated with the highest NIV/IMV utilization rate whereas age >80 years had reduced NIV/IMV utilization. A decreased NIV/IMV usage rate in people older than 80 is notable on account of an increase in DNR (do not resuscitate) orders among patients aged >75 compared with those aged <65, according to a previous meta-analysis [19]. Furthermore, women received more trails of NIV compared to men that was also supported by the Taiwanese study by Shen et al. [20]. Additionally, we found obese patients were at higher odds of receiving ventilation that was even validated by Anzueto et al., and due to an increasing incidence of increased body weight in the general population, it is likely that more overweight patients will require mechanical ventilation in the future [21]. A number of studies have documented a marked increase in respiratory issues, especially respiratory failure in paralysis patients following either spinal cord injury or neuromuscular diseases due to several mechanisms, and the impact of NIV use on those cases [22]. Therefore, it could be an obvious explanation for the increase in ventilation usage in paralysis patients.

Furthermore, uninsured/self-pay patients had lower odds of receiving any sort of ventilation as these patients are more likely to receive the lowest intensity of care such as radiographic and surgical procedures, consultations, and ICU care, which might be the leading contributing factor for lower utilization of invasive or non-invasive ventilation [23-25]. Additionally, our study showed that small and rural hospitals were associated with a lower rate of ventilation utilization that can be attributed to restricted facilities and fewer resources in small and rural hospitals serving as a reason to transfer patients to a greater extent to larger or academic hospitals capable of providing the required definitive care [26]. It is hypothesized that patients treated by a health care provider or admitted to a medical facility on a weekend or holiday might experience worse outcomes than patients treated on weekdays [27]. Proposed reasons for this include a decreased availability of staff or other resources on the weekend, limited access to laboratory or diagnostic tools, or selection bias stemming from a general reluctance by patients to solicit care during the weekend, which we found in our study as well as patients admitted during the weekend who received mechanical ventilation compared to the weekday admissions [27].

This study was able to track the trends of in-hospital mortality and discharge to facility among the three groups in a 10-year period. This can serve as a gateway to conduct different studies into identifying useful tools to decrease mortality and also improve quality of care. The overall in-hospital mortality has decreased in all ventilation groups from 2007 to 2017. This finding was also consistent with 15-year trends in CAP patients that reported a decrease in in-hospital mortality in patients who received non-invasive or invasive ventilation or both [28]. This may be attributed to a better understanding of CAP treatment, adoption of better practice guidelines and care bundles, improved quality of care, and early and/or aggressive treatment. A study conducted by Dean et al. showed that the implementation of a pneumonia practice guideline was associated with a reduction in 30-day mortality among elderly pneumonia patients [29].

The proportion of mortality still remains the highest among those who required IMV. This may be explained by the increased severity of disease in patients where they require invasive ventilation, as evidenced by higher CURB-65 scores, Acute Physiology and Chronic Health Evaluation II (APACHE-II) scores and other predictors, including acute respiratory failure [30]. Overall discharge to facility has been relatively consistent throughout 2008 to 2017 in the respective ventilation groups, while discharge to home has mildly increased in each group, with the highest in the NIV group. This is supported by the studies by Nicolini et al. that concluded that NIV treatment had a high rate of success [31-33]. However, the same studies also concluded the use of NIV in severe CAP as controversial due to greater variability in success compared to other pulmonary conditions. This was echoed by other studies that associated NIV with high failure rates without improvement in mortality [3,34,35]. Further studies into the role of NIV and variables impacting its efficacy in CAP are required. The length of stay at hospital was predictably lowest in those who did not require any ventilation and highest among those who required IMV. This may be attributed to early recovery in those who did not need ventilation. The IMV group typically has more severe disease and likely to have further complications thus extending the length of stay at the hospital.

Our study has several limitations. First, our study is a retrospective study that makes it subjectable to selection bias. Secondly, the selection of samples relies on accurate coding practices that could have confounded our results. Despite its limitations, our study has several strengths. Our study is the first comprehensive nationwide study looking at the noninvasive and invasive ventilation utilization during hospitalization due to CAP and our sample size most closely represents the 95% standardized U.S. population, which reduces the chance of selection bias [5].

## Conclusions

Our study showed that the total hospitalizations due to CAP have trended downwards from 2008 to 2017. Also, there was an increase of NIV use in CAP while the trend of IMV use in CAP has decreased. Furthermore, several predictors associated with an increased NIV/IMV usage rate are age 50-64, septicemia, congestive heart failure, history of chronic pulmonary disease and pulmonary circulatory disease; LOS was also higher among the ventilation groups. As per our study, there are various modifiable factors that can improve the outcomes of the patients admitted with CAP requiring ventilation support and future studies should focus on assuaging the outcomes of patients.
